# Serological Findings in Pigs Vaccinated Against *Actinobacillus pleuropneumoniae* and the Porcine Reproductive and Respiratory Syndrome Virus

**DOI:** 10.3390/vetsci13010091

**Published:** 2026-01-15

**Authors:** Julian Bregen, Nicole de Buhr, Katrin Strutzberg-Minder, Marta C. Bonilla, Rabea Imker, Birte Wegner, Fritjof Freise, Isabel Hennig-Pauka

**Affiliations:** 1 vetxperts Dümmerland, Bahnhofstraße 40, 49439 Steinfeld, Germany; julian.bregen@tiho-hannover.de (J.B.); bwegner@duemmerland.de (B.W.); 2 Institute of Biochemistry, University of Veterinary Medicine Hannover, Foundation, Bünteweg 17, 30559 Hannover, Germany; nicole.de.buhr@tiho-hannover.de (N.d.B.); marta.bonilla.gonzalez@una.cr (M.C.B.); rabea.imker@tiho-hannover.de (R.I.); 3 Research Center for Emerging Infections and Zoonoses (RIZ), University of Veterinary Medicine Hannover, Foundation, Bünteweg 17, 30559 Hannover, Germany; 4 Innovative Veterinary Diagnostics (IVD) GmbH, Albert-Einstein-Straße 5, 30926 Seelze, Germany; 5 Department of Biometry, Epidemiology and Information Processing, University of Veterinary Medicine Hannover, Foundation, Bünteweg 2, 30559 Hannover, Germany; fritjof.freise@tiho-hannover.de; 6 Field Station for Epidemiology, University of Veterinary Medicine Hannover, Foundation, Büscheler Straße 9, 49456 Bakum, Germany; isabel.hennig-pauka@tiho-hannover.de

**Keywords:** ELISA, functional antibodies, opsonophagocytotic assay, vaccination, viremia, survival factor

## Abstract

The reasons for disease outbreaks caused by *Actinobacillus pleuropneumoniae* (APP) in vaccinated pigs are often unknown and remain a challenge for farmers and veterinarians. One hypothesis for APP vaccine failure is the timing of APP vaccination during field or vaccine-induced viremia with Porcine Reproductive and Respiratory Syndrome Virus (PRRSV), which may negatively affect the immune response to APP vaccination. In this study, fattening pigs were vaccinated with a modified live vaccine (MLV) against PRRSV either at the beginning of the fattening period (group G1) or six weeks later (group G2). All pigs were vaccinated against APP five days after the start of fattening, which coincided with MLV-PRRSV viremia in G1. Within both G1 and G2, four subgroups of pigs (*n* = 10) were vaccinated with three different APP vaccines or remained unvaccinated to assess serological responses to various APP antigens. MLV-PRRSV viremia had no significant effect on APP-ApxII (*p* = 0.127), APP-LPS (*p* = 0.120), or opsonophagocytic antibody responses on day 40 of fattening. Lung lesion scores at slaughter were significantly higher (*p* = 0.004) in pigs from G2 (1.82 ± 2.38) compared with those from G1 (0.65 ± 0.88). All APP vaccines elicited presumably protective opsonophagocytic antibodies. In conclusion, no effects of MLV-PRRSV viremia on serological responses following APP vaccination were observed.

## Simple Summary

Vaccination of fattening pigs against the respiratory pathogens *Actinobacillus pleuropneumoniae* (APP) and Porcine Reproductive and Respiratory Syndrome Virus (PRRSV) is common practice. For APP vaccination, inactivated or subunit vaccines are used, whereas modified live vaccines (MLV) are applied for PRRSV vaccination. Despite vaccination against APP, severe disease outbreaks may still occur, and the underlying causes of vaccine failure are often unclear. The hypothesis of this study was that APP vaccination during MLV-PRRSV-induced viremia affects the serological response against APP. However, no differences were observed in serological responses to various APP antigens or in presumably protective opsonophagocytic antibodies between pigs vaccinated against APP with three different vaccines during MLV-PRRSV viremia and pigs vaccinated against PRRSV six weeks after APP vaccination, at a time distant from MLV-PRRSV viremia. Lung lesion scores at slaughter were lower in pigs vaccinated against APP during MLV-PRRSV viremia at the beginning of the fattening period. All APP vaccines elicited antibody responses. In conclusion, this study found no effects of MLV-PRRSV viremia on the serological responses induced by APP vaccination.

## 1. Introduction

Two major respiratory pathogens in pig production are *Actinobacillus pleuropneumoniae* (APP) and the Porcine Reproductive and Respiratory Syndrome Virus (PRRSV), both of which cause substantial health and economic losses on pig farms worldwide [1,2,3]. The primary target cells of PRRSV are differentiated alveolar macrophages; therefore, PRRSV predisposes pigs to secondary infections with various respiratory tract pathogens, frequently resulting in respiratory disease in piglets and fattening pigs [3,4,5].

APP is a Gram-negative, rod-shaped bacterium belonging to the family *Pasteurellaceae* and is the causative agent of porcine pleuropneumonia [1,6]. The disease may follow a peracute course with sudden death, an acute course characterized by severe dyspnea and typical fibrinohemorrhagic and necrotizing lung lesions, or subacute to chronic courses in which lung lesions are detected during slaughterhouse examinations [6,7].

Currently, two biovars comprising a total of 19 serotypes are recognized for APP, which can be differentiated based on distinct capsule synthesis (*cps*) genes [8,9]. The serotypes are characterized by the production of different Apx toxins (ApxI–ApxIV), which vary in their hemolytic and cytolytic activities and are considered important virulence factors [10,11]. Apx toxins I–III can also be produced by other *Actinobacillus* species or members of the *Pasteurellaceae* family [10,12], whereas ApxIV is specific to APP and is expressed by all serotypes during infection in vivo [11,13].

Antibodies against ApxIV are produced exclusively by infected pigs and are not induced by vaccination. Consequently, an ApxIV-based ELISA can be used to differentiate between vaccinated and infected animals and to monitor APP-free herds [10,11,13,14]. Clinically healthy but colonized pigs may harbor APP on their tonsils without inducing ApxIV antibody production [12].

The lipopolysaccharides (LPS) of APP are involved in adhesion to target cells [15,16] and enhance the cytotoxic effects of Apx toxins against phagocytes [15,17]. Following adhesion to host cells, APP releases Apx toxins, resulting in cell lysis [18]. Coinfection with other respiratory pathogens can exacerbate the severity of APP-associated disease [3].

Conflicting results have been reported regarding coinfections with APP and PRRSV. PRRSV is known to suppress host immune defenses, thereby facilitating infection with secondary bacterial pathogens. A predisposition to secondary APP infection caused by PRRSV has been observed in field studies [19,20]. However, experimental challenge studies involving both pathogens have yielded variable results, with reports of either increased severity of lung lesions or no significant changes following APP challenge in pigs previously infected with PRRSV [21]. Notably, the APP strain used in these experiments was considered to be of low virulence. In contrast, in vitro studies have demonstrated antiviral properties of APP, leading to inhibition of PRRSV infection [22].

Vaccination against PRRSV and APP represents an important control strategy to prevent disease outbreaks and to stabilize herd health [7,23]. Nevertheless, severe disease outbreaks may still occur in vaccinated herds. Commercial vaccines against APP in Germany are either inactivated whole-cell vaccines (bacteria) including expressed Apx toxoids I–III or a pure subunit vaccine containing Apx toxoids and OMP antigens [24]. Serological responses following APP vaccination have not been extensively investigated. Pigs vaccinated with commercial vaccines containing ApxI–III have been shown to develop antibodies against these toxins while remaining negative in the ApxIV ELISA [14]. Additionally, APP vaccination can result in positive LPS ELISA responses [25].

Consequently, although serological findings—despite their inherent limitations—may reflect humoral immune responses following APP vaccination or infection, they do not permit definitive conclusions regarding protective immunity.

Vaccination with commercial PRRSV-1 and PRRSV-2 modified live virus (MLV) vaccines induces a viremia of variable duration, depending on the age of the animals and the specific vaccine strain used [26,27]. Understanding the effects of MLV-PRRSV- or PRRSV field strain-induced viremia on the efficacy of simultaneous APP vaccination is of high practical relevance when determining optimal vaccination schedules.

Therefore, the aim of this study was to analyze serological responses following APP vaccination administered during MLV-PRRSV–induced viremia. Serum samples from fattening pigs vaccinated with different commercial APP vaccines were retrospectively examined for antibodies against various APP antigens, as well as for opsonophagocytic antibodies.

## 2. Materials and Methods

### 2.1. Herd Description and Study Population

The study was conducted during a single fattening period on a farm located in a high pig-density region of Lower Saxony, Germany. The farm had a total capacity of 1780 pigs. In this study, 1021 pigs (Danbred x Duroc) were monitored in two stables (G1 and G2).

Pigs were introduced to the farm at 10 weeks of age, with an average body weight of 30 kg, and a high health status from a nursery farm in Eastern Germany, which was considered free of PRRSV, *Mycoplasma hyopneumoniae*, and APP according to routine monitoring protocols of the responsible veterinarian. Although pigs were routinely vaccinated against APP (Coglapix^®^, Ceva Tiergesundheit GmbH, Düsseldorf, Germany), PRRSV (Ingelvac PRRSFlex^®^ EU; Boehringer Ingelheim Vetmedica GmbH, Ingelheim am Rhein, Germany) and Porcine Circovirus 2 (PCV-2) (Ingelvac CircoFLEX^®^, Boehringer Ingelheim Vetmedica GmbH) upon arrival, repeated outbreaks of APP serotype 2–related pleuropneumonia had occurred during fattening at varying intervals in the years prior to the study.

To ensure accurate monitoring of the pigs’ health status and to allow retrospective evaluation in the event of disease outbreaks, individual pigs were permanently identified via ear tags and repeatedly sampled. Five randomly selected pigs from four pens in G1 and four pens in G2 (*n* = 40) were sampled at arrival (day 0), on day 5, and on day 40 of fattening. Serum samples were stored in a biobank until slaughter, allowing retrospective serological analysis of paired or triplicate samples if disease outbreaks occurred. This preventive diagnostic approach was recommended by the veterinarian due to recurrent PRRSV and APP outbreaks in previous years. Negative serological results at arrival confirmed the health status of the farm of origin. Day 5 samples allowed detection of acute PRRSV infection potentially acquired during transport or introduced via vehicles or air after arrival, while day 40 samples enabled reconstruction of the timing of infection.

**Figure 1:**
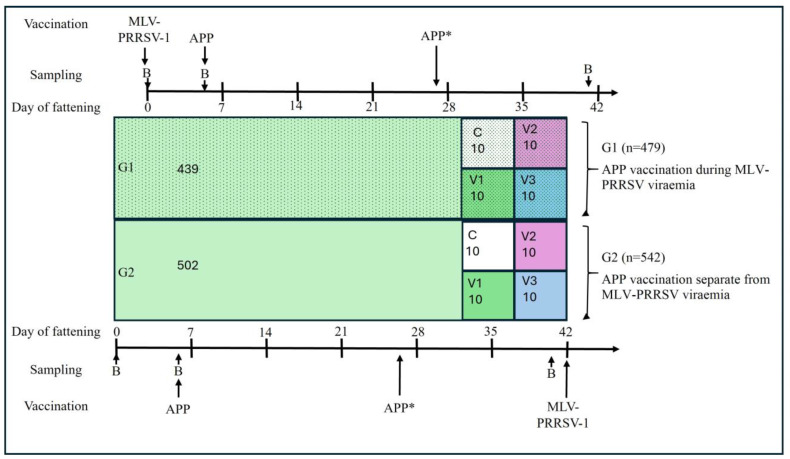
Pig groups, vaccination and sampling schemes Pigs for stable G1 (*n* = 479) were delivered to the fattening farm four days after delivery of pigs for stable G2. Pigs in G1 were vaccinated at arrival (day 0 of fattening) against PRRSV, while pigs in G2 were vaccinated against PRRSV six weeks later (day 42 of fattening). Within each stable four pens with ten pigs each are four different subgroups with either pigs not vaccinated against APP serving as controls (C, white boxes), vaccinated with Coglapix^®^ (V1, green boxes), vaccinated with Suivac^®^ (V2, pink boxes) or vaccinated with Porcilis^®^ (V3, blue boxes). Only V1–V3 received a second vaccination against APP three weeks after the first vaccination (APP*). Remaining pigs in G1 (*n* = 439) and G2 (*n* = 502) were vaccinated only once against APP with Coglapix^®^ on day 5 of fattening. Blood from five animals out of subgroups C and V1–V3 were taken at three different time points (B).

For the field study, the vaccination protocol was adapted with respect to APP and PRRSV. Pigs for stable G1 were delivered four days after pigs for stable G2 for logistical reasons (Figure 1). Two different PRRSV vaccination time points were implemented: 479 pigs in G1 were vaccinated against PRRSV immediately upon arrival, while 542 pigs in G2 were vaccinated six weeks later, deviating from the routine protocol (Figure 1). All pigs were vaccinated against APP five days after arrival with a vaccine containing APP serotype 1 and 2 bacterins and Apx toxins I–III (Coglapix^®^), with the exception of 60 pigs: In both stables, pigs in three pens were either unvaccinated (control), vaccinated with a commercial APP vaccine containing APP serotypes 2 and 9 bacterins and Apx toxins I–III (Suivac APP, ChemVet dk A/S, Silkeborg, Denmark), or vaccinated with a commercial APP subunit vaccine containing ApxI–III and an outer membrane protein (Porcilis^®^ APP, Intervet Deutschland GmbH, Unterschleißheim, Germany) (Figure 1). According to this schedule, APP vaccination in stable G1 occurred during MLV-PRRSV viremia.

In four pens with ten pigs each in G1 and G2, a second APP vaccination was administered three weeks after the first. All other pigs in both stables did not receive a booster vaccination. All vaccines were administered intramuscularly. Animals were handled according to high ethical standards and national legislation, and unnecessary suffering was minimized through appropriate precautions during sampling and examination. Blood sampling was performed by an experienced veterinarian, and pigs were continuously monitored until slaughter.

### 2.2. Clinical and Slaughterhouse Examinations

In addition to documenting diseased and deceased animals, a cough and sneeze index was recorded weekly at the pen level in G1 and G2 by the same observer [28]. Pigs in each pen were encouraged to stand up by calling and clapping. The number of coughs and sneezes was then counted over a 2 min period. A second cough from the same animal was only recorded if at least 10 s had passed since the previous cough. The cough and sneeze indices were calculated by dividing the total number of coughs or sneezes by the number of pigs in the pen and by the 2 min observation period, resulting in the mean number of coughs or sneezes per pig per minute in the respective pen [28,29].

Slaughterhouse lung examinations were performed on randomly selected pigs by the same examiner (G1: *n* = 54 and G2: *n* = 74), excluding pens with subgroups C and V1–V3. The severity of lung lesions was assessed using the Ceva Lung Program (CLP^®^, Ceva Tiergesundheit GmbH, Düsseldorf, Germany), which is based on a modified Madec-Kobisch Score, in combination with the Slaughterhouse Pleurisy Evaluation System (SPES) score for assessment of presence, extent and location of pleuritis, assigning points from 0 to 4 [20,30,31].

### 2.3. Routine Laboratory Diagnostic Examinations

Triplicate serum samples stored in the veterinary practice’s internal biobank were used for retrospective laboratory diagnostic testing. Blood samples were centrifuged at 2000× *g* for 10 min on the day of collection, and the serum was subsequently frozen in Eppendorf tubes at −20 °C in the biobank until serological testing.

Serum samples collected at arrival (day 0) and on day 40 of the fattening period were tested in commercial ELISA test kits for ApxIV-antibodies (IDEXX APP-ApxIV Ab Test^®^, IDEXX Laboratories Inc., Westbrook, ME, USA) and APP-LPS-antibodies (ID Screen APP Screening indirect, IDVet, Grabels, France) according to the manufacturer’s instructions. ApxII-antibodies were determined as described by Leiner et al. [32]. Serum samples collected on day 5 of fattening were tested for the presence of PRRSV viremia using a commercial real-time RT-PCR assay (EZ-PRRSVTM MPX 4.0 RT-PCR Target Specific Reagents for the rapid Identification and Differentiation of North American and European PRRSV Viral RNA, Tetracore Inc., Rockville, MD, USA) following the manufacturer’s instructions.

### 2.4. Opsonophagocytotic Assay

An opsonophagocytotic assay established for scientific purpose at the Institute of Biochemistry of the University of Veterinary Medicine Hannover was used to measure functional pathogen-specific antibodies in serum samples collected on days 0 and 40 of the fattening period by assessing antibody-induced opsonophagocytic killing of the pathogen [33,34,35].

The principle of this assay is the quantification of the effect of pathogen-specific functional antibodies that enable neutrophils to eliminate the pathogen through phagocytosis. The assay requires blood cells of a healthy donor pig. These cells were separated from plasma, purified by washing, and subsequently reconstituted with serum from the pigs to be tested. Comparability of the survival factor (SF) between different serum samples is only ensured when cells from the same donor pig are used.

In the present study, two different donor pigs were required due to the large number of samples. Cells from one donor pig were used to compare APP vaccine subgroups (C, V1–V3) in G1, while cells from the second donor pig were used to compare APP vaccine groups (C, V1–V3) in G2. An APP strain was added to the reconstituted blood and incubated for 2 h at 37 °C. Colony forming units (CFU) were quantified by plating serial dilutions at the start and end of the incubation period onto agar plates, followed by overnight cultivation at 37 °C in a 5% CO_2_ atmosphere. The APP serotype 2 field strain C3656/0271/11 was used for inoculation [36]. The SF for each serum sample was calculated as the ratio of CFU/mL after 2 h of incubation to CFU/mL at the start of incubation. A SF < 1 indicates effective pathogen elimination and reflects the presence of specific opsonophagocytotic antibodies in the respective serum.

### 2.5. Statistical Evaluation

All laboratory and clinical data were recorded in Microsoft^®^ Excel, version 16.75 (Microsoft Corporation, Redmond, WA, USA) for data management and graphing. Statistical analyses were performed using SAS software, version 9.4, and SAS Enterprise Guide, version 7.15 (SAS Institute, Cary, NC, USA).

Lung scoring results were compared between G1 and G2 using the Wilcoxon test. Spearman’s correlation coefficients were calculated to test for significant correlations between different APP antibody levels on day 40 of the fattening period.

ApxII-antibody levels at day 40 of fattening were modeled using a Poisson regression model with PRRSV viremia status and APP vaccine as main effects, including their two-factor interaction.

LPS-antibody levels were analyzed using a separate Poisson regression model with PRRSV-viremia, APP vaccination, and time point as main effects. All two factor interactions and the three-factor interaction of the main effects were included, along with a random effect to account for repeated measurements from the individual pig at the two time points.

Both Poisson models were fitted using PROC GLIMMIX (SAS), the log-link and standard estimation techniques (maximum likelihood for ApxII and residual pseudo-likelihood for LPS). They included a multiplicative dispersion parameter to assess possible over-dispersion. This parameter was estimated as Pearson Chi-Square/DF. An estimate > 1 indicated over-dispersion in both cases.

For the survival factor (SF) a linear mixed model was fitted to the natural logarithm of the SF assuming a Gaussian response distribution and identity link function using restricted maximum likelihood (PROC GLIMMIX). The same main effects and interactions as used for the LPS antibody analysis were included, together with a random effect to account for repeated measurements for the individual pig over time. Distributional assumptions were checked graphically using the residuals.

Post hoc tests were adjusted using the Tukey–Kramer method. A significance level of 0.05 was applied.

## 3. Results

### 3.1. Clinical Findings

Animal data are summarized in Table 1. The good health status of all the pigs was demonstrated by the minimal clinical signs (sneezing and coughing) they displayed throughout the fattening period. There were no outbreaks of PRRSV or APP during the fattening phase. Both G1 and G2 mortality rates were less than 1%. The lameness of ten individual fattening pigs in both groups required the use of non-steroidal anti-inflammatory drugs (NSAIDs) and penicillin. A maximum of 1% of coughing pigs per minute was observed in both G1 and G2, indicating persistently low cough indices. The sneeze indices were also low with a maximum of 2% sneezing pigs per minute.

**Figure 2:**
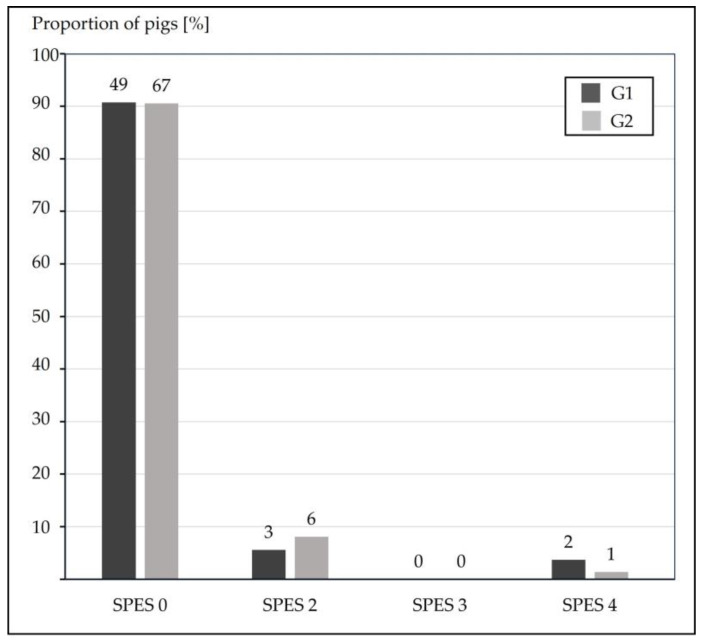
Relative frequencies of dorsocaudal pleurisy. Dorsocaudal pleurisy in both groups (G1: *n* = 54, G2: *n* = 74) scored as SPES 0: no lesion; SPES 2: a dorsocaudal, monolateral focal lesion; SPES 3: a dorsocaudal, bilateral focal lesion or extended monolateral lesion (at least 1/3 of one diaphragmatic lobe); SPES 4: a severely extended bilateral lesion (at least 1/3 of both diaphragmatic lobes). Absolute numbers are depicted above the bars.

**Figure 3:**
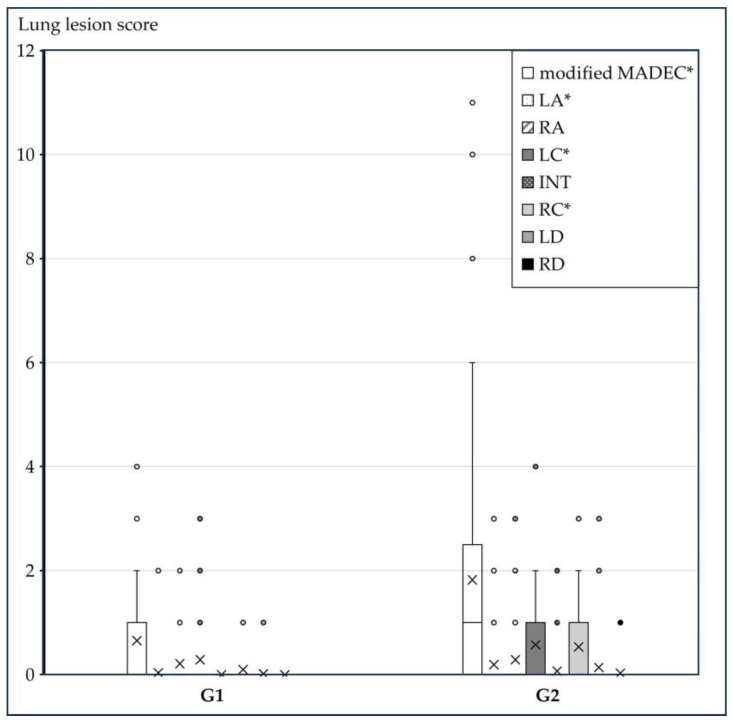
Lung lesions in different lung lobes. Lung lesion scores in both groups (G1: *n* = 54, G2: *n* = 74) are shown. From left to right per group: modified MADEC: total lung sum score, LA: left apical lobe, RA: right apical lobe, LC: left cardiac lobe, INT: intermediate lobe, RC: right cardiac lobe, LD: left diaphragmatic lobe, RD: right diaphragmatic lobe. Boxes represent the interquartile data (50% between 25% and 75% quartiles) of lung scores. The line inside the boxes indicates the median. The upper fences represent maximal values without outliers. The crosses represent the mean values. The small dots represent outliers. Significant differences between G1 and G2 are indicated by asterisks.

A total of 128 lungs were examined at the slaughterhouse, 54 from G1 and 74 from G2 (no pigs of subgroups C, V1–V3 included). Dorsocaudal pleurisies were found in less than 10% of the lungs analyzed, with no differences between the two groups (G1 9.3% vs. G2 9.5%) as shown in Figure 2. The modified Madec lung lesion scores resulted in a significantly higher (*p* = 0.004) score in pigs of G2 (1.82 ± 2.38) than G1 (0.65 ± 0.88) as shown in Figure 3.

### 3.2. Serological Findings

PRRSV viremia was detected on day 5 of fattening in all 20 examined pigs in G1, which had been vaccinated against PRRSV-1 on day 0 of fattening, while all 20 examined pigs from G2 were negative (Table 1).

**Figure 4:**
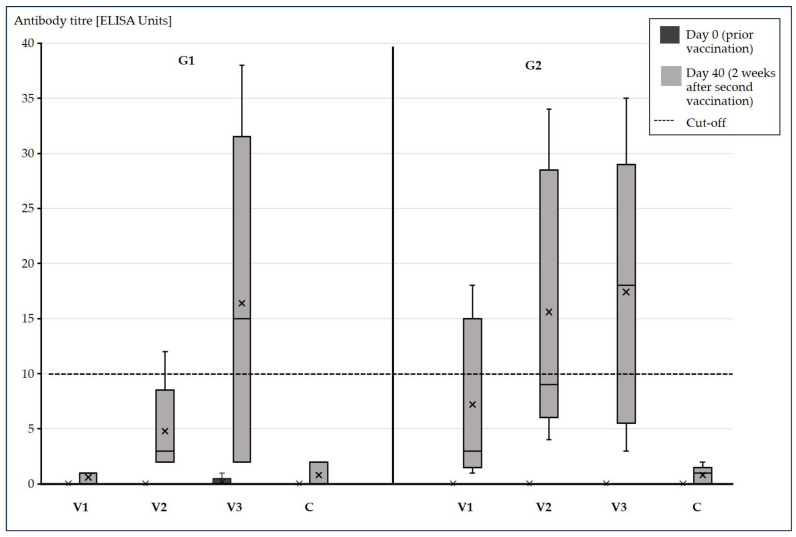
Antibodies against the APP-ApxII toxin. Antibodies against the APP-ApxII toxin within the different PRRSV-vaccinated groups (G1: PRRSV viremia, G2: no PRRSV viremia at the time point of the first APP vaccination at day 5 of fattening) at day 0 of fattening (prior APP vaccination) and 2 weeks after the second APP vaccination at day 40 of fattening. The cut-off value of 10 is indicated by a dotted line. Vaccination groups V1–V3 and the control group C contained 5 pigs in G1 and 5 pigs in G2, respectively. Boxes represent the interquartile data (50% between 25% and 75% quartiles) of ELISA units. The line inside the boxes indicates the median. The upper and lower fences represent maximal and minimal values. The crosses represent the mean values.

**Figure 5:**
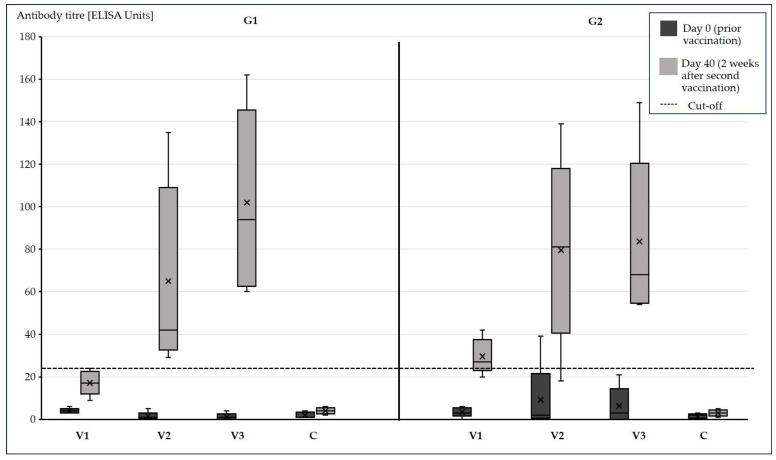
Antibodies against the APP-LPS. Antibodies against APP-LPS within the different PRRSV-vaccinated groups (G1: PRRSV viremia, G2: no PRRSV viremia at the time point of the first APP vaccination at day 5 of fattening) at day 0 of fattening (prior APP vaccination) and 2 weeks after the second APP vaccination at day 40 of fattening. The cut-off value of 25 is indicated by a dotted line. Vaccination groups V1-V3 and the control group C contained 5 pigs in G1 and 5 pigs in G2, respectively. Boxes represent the interquartile data (50% between 25% and 75% quartiles) of ELISA units. The line inside the boxes indicates the median. The upper and lower fences represent maximal and minimal values. The crosses represent the mean values.

All 40 examined pigs from both groups G1 and G2 were tested negative in the ApxIV- and ApxII-ELISA on day 0 of fattening, while one pig from G2 V2 was positive in the APP-LPS-ELISA at this time point (Figure 4 and Figure 5). Although they were all below the corresponding ELISA cut-offs, APP-LPS- and -ApxII-antibodies were also detected in other fattening pigs at this time. The ApxII-ELISA and the LPS-ELISA produced positive results on day 40 of fattening, but no ApxIV antibodies were found (Table 2, Figure 4 and Figure 5). While the APP vaccine (V1–V3) had a significant effect (*p* = 0.001) on ApxII-antibody levels on day 40 of fattening, the early PRRSV vaccination on day 0 of fattening had no significant effect (*p* = 0.127), including the interaction (*p* = 0.220). Antibody levels were higher in pigs vaccinated with V3 than in pigs vaccinated with V1 (adjusted *p* = 0.045) and non-vaccinated pigs (C) (adjusted *p* = 0.009). The PRRSV vaccination (*p* = 0.120) and its two-factor interactions with the APP vaccine (*p* = 0.371) and the time point (*p* = 0.109) had no significant effect on LPS antibody levels. The same was true for the three-factor interaction of all factors (*p* = 0.061). The APP vaccine (*p* = 0.0002), the time point itself (*p* < 0.0001) and their interaction (*p* = 0.0006) were statistically significant with respect to the LPS antibodies. There was no significant difference between the vaccination groups at day 0 of fattening (all adjusted *p* > 0.9). The LPS antibody levels increased over time in all APP vaccinated groups (adjusted *p* < 0.001). There was no significant change in the control group (adjusted *p* = 0.921). On day 40 of fattening differences between C and the other groups as well as differences between V1 and the other vaccinated groups V2 and V3 were statistically significant (adjusted *p* < 0.001). The difference between V2 and V3 was not significant (adjusted *p* = 0.837).

Mean values and standard deviations of ApxII-, LPS-antibodies and survival factors (SF) of 5 pigs in the different vaccination groups (V1–3) and non-vaccinated group (C) on day 0 and 40 of fattening. Cut-off values of the different ELISA: ApxII-ELISA: 10; LPS-ELISA: 25; SF: 1.

### 3.3. Opsonophagocytotic Assay Results

**Figure 6:**
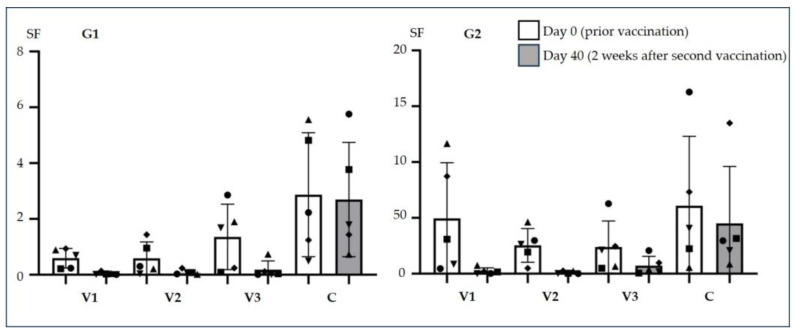
Survival factors (SF) as results of the opsonophagocytotic assay. Individual SF of the differently vaccinated animals within the two groups (G1: PRRSV viremia, G2: no PRRSV viremia) prior to vaccination on day 0 of fattening (prior vac) and 2 weeks after the second vaccination on day 40 of fattening (after vac). Bars indicate mean values and standard deviations. Vaccination groups V1–V3 and the control group C contained 5 pigs in G1 and 5 pigs in G2, respectively. Each symbol in each vaccination group represents the same pig in this respective group.

In the SF analysis PRRSV status (*p* = 0.003), timepoint (*p* < 0.0001), APP vaccination group (*p* < 0.0001) and the interaction between APP vaccination and time (*p* = 0.0002) were statistically significant. The SF against the APP serotype 2 strain used was high in serum from all pigs taken at day 0 of the fattening period, prior to APP vaccination (Figure 6), and was lower in sera from APP vaccinated pigs on day 40 of fattening, which was two weeks after the second APP vaccination. The change in mean SF was significant in pigs vaccinated in V1 (adjusted *p* < 0.0001), V2 (adjusted *p* < 0.0001) and V3 (adjusted *p* = 0.002). In the control group, no significant change in SF between days 0 and 40 of fattening was observed. Although no significant differences were found among the four groups at day 0 of fattening, and no significant differences were observed among groups V1, V2, and V3 at day 40 of fattening (adjusted *p* > 0.371), the mean SF of each vaccinated group differed significantly from that of the control group (V1 adjusted *p* < 0.0001; V2 adjusted *p* < 0.0001; V3 adjusted *p* = 0.0003), with vaccinated groups showing lower mean SF values than the control.

On day 40 of fattening LPS antibody levels were significantly correlated with ApxII antibody levels (r_s_ = 0.77, *p* < 0.0001) and were negatively correlated with SF (r_s_ = −0.46, *p* = 0.0026).

## 4. Discussion

The aim of this study was to assess (i) serological responses after vaccination with different APP vaccines and (ii) the potential effects of MLV-PRRSV viremia on serological responses after APP vaccination.

The high prevalence of APP in conventional farms in the swine-dense region in northwestern Germany has been described previously [37], indicating that APP-naïve pigs transported into this region are at high risk of infection. Independent of the immune status of pigs introduced into a farm, general risk factors for outbreaks of respiratory disease include large herd sizes, high stocking density, short distances between neighboring farms, and frequent animal transport and trade connections [37,38]. The fattening farm included in this study was selected because of recurrent APP outbreaks over several years, which may have been partly related to the immune status of naïve pigs introduced to the farm. During the studied fattening period, ApxIV ELISA results remained negative until day 40 of fattening, and no clinical signs were observed until the end of the fattening period. Although pigs were not sampled again at the end of fattening, it can be assumed that they were not infected with APP during this period. Typically, ApxIV antibodies become detectable by ELISA approximately three weeks after APP infection [14]. The absence of clinical signs, the low mortality rate throughout the fattening period, and the low number of APP-typical lung lesions further support the absence of APP infection at slaughter, even though the pigs were naïve at arrival. Whether this was also the case for PRRSV cannot be confirmed, as strains of lower virulence may not cause disease, particularly in pigs vaccinated against PRRSV.

Antibodies against ApxII were detected in one pig prior to the first APP vaccination, although levels were below the assay cut-off. These pre-vaccination ApxII antibodies may have been induced by other *Actinobacillus* species, such as *Actinobacillus* (*A.*) *rossii*, *A. suis*, or *A. porcitonsillarum*, which are also known to produce ApxII toxin [10,12,39]. Similarly, the presence of pre-vaccination APP-LPS antibodies in some animals may be attributable to cross-reactivity with other pathogens [40].

Different serological responses measured by ELISA following vaccination with different App vaccines have been reported previously [41]. In those studies, pigs vaccinated with the commercial vaccines V1 and V3 showed increased antibody levels in the respective ELISAs, which is consistent with the observations of the present study.

In our study, APP-LPS antibodies were correlated with functional antibodies, as reflected by the SF.

Overall, animals in this study showed low morbidity, mortality, and pleurisy rates at slaughter. The sneeze and cough indices were also very low. The cough index reached a maximum value of 1% and thus remained below the threshold of 2.5% for coughing pigs, which is considered indicative of *M. hyopneumoniae* infection [28]. All APP vaccines used in this study have been proven effective in challenge experiments conducted during vaccine approval as well as in field trials [20,42,43,44,45]. Apx toxins are highly immunogenic antigens, and their necessity for immunization has been demonstrated, indicating that secreted Apx toxins are required components of APP vaccines [24,46]. However, although Apx toxins are essential vaccine components, they are not solely responsible for the protective effect, and multiple antigens are required to achieve effective protection [47,48,49]. All commercial vaccines used in this study were developed based on these principles but differed in their composition. The use of commercial APP vaccines has been shown to reduce not only APP-typical lesions but also the number of lungs affected by non-specific bronchopneumonia [20]. This effect may reflect a beneficial impact of APP vaccination through a reduction in APP infection and, consequently, in secondary bacterial infections. More specifically, APP vaccination has been associated with a reduction in mycoplasma-like pneumonia in addition to a decrease in APP-typical lung lesions [44].

Unexpectedly, lung lesion scores differed significantly between G1 and G2, with higher scores observed in pigs vaccinated against PRRSV on day 42 of fattening. Pigs in G2 were housed in a different stable that was identical in construction to the stable G1; however, air flow, climate conditions, and infection dynamics involving pathogens other than PRRSV and APP may have differed between the two stables.

Several confounding factors that cannot be controlled under field conditions may account for the observational finding that pigs vaccinated against PRRSV on day 0 of the fattening period exhibited the lowest lung lesion scores at slaughter. It cannot be excluded that pigs were exposed to a PRRSV field strain at a later stage, in which case pigs vaccinated at day 0 of the fattening period may have been better protected than those vaccinated on day 42. Although PRRSV vaccination does not prevent infection, it reduces shedding of field virus [32]. The peak of PRRSV-specific neutralizing antibodies occurs approximately four weeks after PRRSV-MLV vaccination, while PRRSV-specific cell-mediated immune responses develop between weeks 2 and 4 post-vaccination [26]. If pigs in group G1 were better protected against lateral transmission of a PRRSV field strain, it is likely that this infection event occurred after the first third of the fattening period. This scenario is plausible based on published data from the United States, where more than half of farms with PRRSV-negative pigs at placement become PRRSV-positive before marketing due to substantial biosecurity challenges [2]. In addition to direct transmission via infected pigs, several indirect transmission routes have been described, including transport vehicles, fomites (clothing, boots, needles, and other equipment), insects, and feed [2]. PRRSV-MLV vaccination of pigs at risk of exposure to field virus strains, particularly in pig-dense areas, has been associated with significant performance advantages [2]. Therefore, it can be assumed that vaccination may reduce the proportion of pigs developing lung lesions. An alternative, though less likely, explanation is non-specific immune stimulation induced by PRRSV vaccination, which could increase resistance to other pathogens. However, evidence for such non-specific vaccine effects is limited, and only negative non-specific effects have been reported for PRRSV-MLV vaccination [50,51]. Specifically, a detrimental non-specific effect on lung lesions caused by *M. hyopneumoniae* has been attributed to the induction of regulatory T cells by the PRRSV vaccination, resulting in an inadequate interferon-γ response [51].

In general, potential non-specific vaccine effects may be influenced by several factors, including the vaccine strain, the use of adjuvants, the route of administration, and the presence of maternal immunity [50].

PRRSV infection itself is associated with impaired local cellular immunity, rendering pigs more susceptible to secondary infections, including *M. hyopneumoniae*, *Glaesserella parasuis*, and *Streptococcus suis* [2,51]. Taken together, the most probable explanation for lower lung lesion scores observed in G1 is exposure to a PRRSV field strain during the fattening period.

Neutrophils are key effector cells of the innate immune system [52]. They are activated by signals following microbial invasion and are capable of binding and eliminating pathogens [53]. One of their major antimicrobial mechanisms is phagocytosis, during which IgG-opsonized pathogens are engulfed into vacuoles and phagosome maturation is initiated [53,54]. During this process, phagosomes acquire the capacity to destroy the pathogen [54]. Vaccine-induced protection may, in part, be reflected by a low survival factor measured in the opsonophagocytotic assay, although this relationship has not yet been confirmed in infection experiments. It can be assumed that sera containing high levels of opsonophagocytic antibodies, or antibodies with high affinity for the pathogen, are more effective in mediating APP killing by neutrophils, resulting in a low survival factor. A limitation of this assay system is its dependence on blood cell donor pigs, which can individually influence assay outcomes. This variability is reflected by generally lower survival factors observed in all sera from pigs in G1, which were mixed with cells from a different donor pig than those used for sera from pigs in G2. Importantly, the killing of APP in sera from non-vaccinated pigs cannot be attributed solely to specific APP antibodies but is also mediated by other neutrophil defense mechanisms, such as the generation of reactive oxygen species (ROS), degranulation, and the formation of neutrophil extracellular traps (NETs) [52,53].

Phagocytosis of APP by alveolar macrophages (AMs) can result in AM death due to the action of Apx toxins, even in the presence of Apx-specific antibodies [18]. In contrast, neutrophils are protected from Apx toxin-mediated cytotoxicity by Apx antibodies and are therefore able to eliminate the pathogen through phagocytosis [55].

In this study, SFs were high in serum samples collected prior to vaccination on day 0 and in the control group, indicating that the pathogen was able to proliferate in serum. Thus, other neutrophil killing mechanisms may not have efficiently eliminated APP, possibly because APP can survive in the presence of NETs [56]. In contrast, SFs were low in serum collected two weeks after the second vaccination on day 40 of fattening, indicating that opsonophagocytotic antibodies against APP had been induced by vaccination, regardless of whether pigs were vaccinated during MLV-PRRSV viremia or not.

It should be noted that the number of animals included in the opsonophagocytotic assay was limited. In addition, SFs may have been influenced by the age of the animals, reflecting increased maturation of the innate immune system in older pigs. This is supported by the lower SFs measured in serum collected after the second APP vaccination on day 40, when pigs were approximately six weeks older than at the time of the first vaccination. Notably, lower SFs were also observed in several unvaccinated control pigs at this later time point.

The complex interactions between different microorganisms and immune responses within swine herds, and their potential impact on vaccine efficacy, remain poorly understood. This study represents an initial attempt to generate hypotheses regarding the influence of viral infection on immune responses following APP vaccination.

Only one MLV PRRSV strain was used in this study, and the results may have differed if another MLV PRRSV strain or a PRRSV field strain had been applied, as different PRRSV strains are known to induce distinct immune responses [57]. Furthermore, no APP outbreak occurred during the study period; therefore, key clinical outcomes such as mortality and disease severity could not be compared between groups. Addressing these limitations will require future experimental studies under standardized conditions with controlled infectious doses.

## 5. Conclusions

Different commercial APP vaccines induced slightly different serological responses as measured by the ApxII- and APP-LPS ELISAs, whereas no differences were observed in functional opsonophagocytotic antibody responses. No interaction between vaccine-induced PRRSV viremia and the serological response to APP vaccination was detected. Pigs with and without vaccine-induced PRRSV viremia developed opsonophagocytotic antibodies and remained in good health throughout the entire fattening period following APP vaccination.

In conclusion, this study mainly characterizes serological immune responses following APP vaccination in PRRSV-1-MLV-viremic and -non-viremic pigs. The absence of an APP infection during the study period precludes assessment of vaccine-induced clinical protection. Vaccine protectivity cannot be inferred from ELISA results obtained in routine diagnostic assays. Consequently, the results should be interpreted as hypothesis-generating, and future studies, including controlled infection models or field outbreaks, are required to confirm the relevance of the serological findings.

## Figures and Tables

**Table 1:** Overview of diagnostical parameters of both groups (G1, G2).

Clinical Examination in All Pigs in G1 and G2	G1	G2
Number of pigs	479	542
Diseased animals (*n*)	4	6
Died animals (*n*)	3	2
Died animals (%)	0.63	0.37
Coughing index mean (%)	0.08	0.12
Coughing index max. (%)	0.85	1.06
Sneezing index mean (%)	1.11	1.08
Sneezing index max. (%)	2.01	1.76
**Slaughterhouse examination in selected pigs** **(no pigs of C, V1–V3)**	**G1**	**G2**
Number of lungs examined at slaughterhouse (*n*)	54	74
Lung lesion score [30], mean ± standard deviation	0.65 ± 0.88	1.82 ± 2.38
**Serological examination in Subgroups C, V1–V3**	**G1**	**G2**
PRRSV viremia at first APP vaccination (positive/total samples)	20/20	0/20
APP ApxIV antibodies, fattening day 0 (positive/total samples)	0/20	0/20
APP ApxIV antibodies, fattening day 40 (positive/total samples)	0/20	0/20

**Table 2:** Serological findings in different ELISA.

Group	Vaccine	Day of Fattening			Day 40 of Fattening		
		ApxII	LPS	SF	ApxII	LPS	SF
G1	V1	0 ± 0	4 ± 1.22	0.59 ± 0.35	0.6 ± 0.55	17.2 ± 5.76	0.05 ± 0.05
	V2	0 ± 0	1.6 ± 1.95	0.59 ± 0.59	4.8 ± 4.21	65 ± 44.41	0.09 ± 0.09
	V3	0.2 ± 0.45	1.2 ± 1.64	1.36 ± 1.17	16.4 ± 15.47	102 ± 43.38	0.18 ± 0.31
	C	0 ± 0	2 ± 1.41	2.87 ± 2.22	0.8 ± 1.10	4 ± 1.58	2.70 ± 2.05
G2	V1	0 ± 0	3.4 ± 2.30	4.96 ± 4.99	7.2 ± 7.46	29.6 ± 8.32	0.23 ± 0.30
	V2	0 ± 0	9.2 ± 16.72	2.54 ± 1.51	15.6 ± 12.54	79.6 ± 44.45	0.12 ± 0.12
	V3	0 ± 0	6.4 ± 8.79	2.40 ± 2.33	17.4 ± 12.62	83.6 ± 39.64	0.73 ± 0.83
	C	0 ± 0	1.6 ± 1.14	6.10 ± 6.22	0.8 ± 0.84	3 ± 1.58	4.51 ± 5.10

## Data Availability

The original contributions presented in this study are included in the article. Further inquiries can be directed to the corresponding author.

## Abbreviations

The following abbreviations are used in this manuscript:

AMs	Alveolar macrophages
APP	*Actinobacillus pleuropneumoniae*
C	Control
CFU	Colony Forming Unit
cps	Capsule synthesis genes
G	Group
LPS	Lipopolysaccharides
MLV	Modified live virus
NETs	Neutrophil extracellular traps
NSAID	Non-steroidal anti-inflammatory drugs
OMPs	Outer membrane protein
PCV-2	Porcine Circovirus 2
PRRSV	Porcine Reproductive and Respiratory Syndrome Virus
ROS	reactive oxygen species
SF	survival factor
SPES	Slaughterhouse Pleurisy Evaluation System
